# Supplementary material for: Sterol regulatory element binding transcription factor 1 promotes proliferation and migration in head and neck squamous cell carcinoma
Source: PeerJ. 2023 Apr 17;11:e15203. doi: 10.7717/peerj.15203 (PMC10117388; doi:10.7717/peerj.15203)
Supplement: Figure S1 [file peerj-11-15203-s001.pdf]

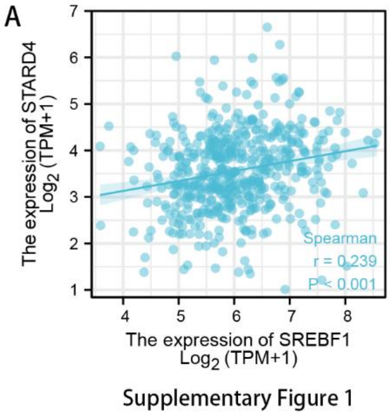

Supplementary Figure 1.Correlation analysis of SREBF1 and STARD4 in the HNSC cohort of TCGA database
